# Natural Fractals as Irreversible Disorder: Entropy Approach from Cracks in the Semi Brittle-Ductile Lithosphere and Generalization

**DOI:** 10.3390/e24101337

**Published:** 2022-09-22

**Authors:** Patricio Venegas-Aravena, Enrique G. Cordaro, David Laroze

**Affiliations:** 1 Department of Structural and Geotechnical Engineering, School of Engineering, Pontificia Universidad Católica de Chile, Vicuña Mackenna 4860, Macul, Santiago 8331150, Chile; 2 Observatorios de Radiación Cósmica y Geomagnetismo, Departamento de Física, FCFM, Universidad de Chile, Casilla 487-3, Santiago 8370415, Chile; 3 Facultad de Ingeniería, Universidad Autónoma de Chile, Pedro de Valdivia 425, Santiago 7500912, Chile; 4 Instituto de Alta Investigación, CEDENNA, Universidad de Tarapacá, Casilla 7D, Arica 1000000, Chile

**Keywords:** fractal cracks, pre-earthquake dynamics, entropy increase, seismo-electromagnetic theory, non-reversible process

## Abstract

The seismo-electromagnetic theory describes the growth of fractally distributed cracks within the lithosphere that generate the emission of magnetic anomalies prior to large earthquakes. One of the main physical properties of this theory is their consistency regarding the second law of thermodynamics. That is, the crack generation of the lithosphere corresponds to the manifestation of an irreversible process evolving from one steady state to another. Nevertheless, there is still not a proper thermodynamic description of lithospheric crack generation. That is why this work presents the derivation of the entropy changes generated by the lithospheric cracking. It is found that the growth of the fractal cracks increases the entropy prior impending earthquakes. As fractality is observed across different topics, our results are generalized by using the Onsager’s coefficient for any system characterized by fractal volumes. It is found that the growth of fractality in nature corresponds to an irreversible process.

## 1. Introduction

Earthquakes are complex natural phenomena that can be studied by using different approaches. Some of them are focused on fault mechanics. That is, the dislocation and rupture of preexisting faults within the lithosphere which generate seismic radiation [1]. Others are focused on the geodetic deformation of the Earth’s surface due the tectonic plates’ drift which generate long-term stress accumulation [2]. Finally, a recent focus corresponds to the electromagnetic signals that can be linked to crack and microcrack generation within the lithosphere which could be considered as the manifestation of impending earthquakes [3]. 

The first approach makes impossible the forecast of major earthquakes due to two main reasons: the vanishing seismic source information due to the attenuation of seismic waves [4] and, the interplay among different heterogeneous physical processes that make the rupture chaotic [5]. Despite this, it has been recently shown that any prediction should be done using multidisciplinary precursors [6]. In that sense, the seismo-electromagnetic approach could be considered as multidisciplinary because is based on experimental studies on brittle lithospheric rock samples, magnetic data and physical analysis. These studies can be summarized with the following five points: (1) the volume of cracks increases before the macroscopic failure [7,8], (2) the external mechanical loads generate detectable electromagnetic signals known as pressure stimulated currents (PSC) [9], (3) electromagnetic signals generated from cracks are well described by the dislocation process known as motion of charged edge dislocations (MCD) [10,11,12,13], (4) the number of cumulative magnetic anomalies near and prior impending megathrust earthquakes also increases [14,15,16], and (5) fractally distributed cracks within the lithosphere could explain and link the above mentioned points in the so-called seismo-electromagnetic theory [17]. 

The physical basis of the seismo-electromagnetic theory is focused on electromagnetic signals that rise due to pre-failure states such as the material cracking or complex multiparametric statistical approach [18,19]. In addition, the theory also takes into account the concept of “earthquake entropy” which relates the lithospheric stress changes to the b-value of the Gutenberg–Richter’s law within faults [20]. That is, a fault’s properties give information about stress states within the lithosphere. In other words, stress states within the lithosphere can be linked to frictional properties which could be related to other seismic parameters such as seismic magnitude or seismic moment [21]. As lithospheric stress generates the fractally distributed cracks and earthquakes correspond to an irreversible process [22], it is expected that the physical process that generates fractal cracks also increases the entropy of the lithosphere before impending earthquakes. The entropy change for one single crack is well understood in terms of electrical current, friction, and temperature [23]. Nevertheless, realistic cracks are fractally embedded within materials. 

On the other hand, it has been shown that other systems similar to fractal cracks evolving in time are also present in several natural phenomena. For instance, some of those systems are (including seismology studies):

- Earthquakes spatial distributions [24],
- Earthquake slip patterns [25],
- Cracks in rocks and lithosphere [17,26],
- Structural geology [27],
- Galaxies clustering [28],
- Self-organized criticality (SOC) systems [29],
- Quantum scale properties [30,31],
- High energy collisions data [32],
- Fractal electrodynamics [33],
- Fractal structures of spacetime and mass [34],
- Snowflakes dendrites distribution [35],
- Biological structures [36,37],
- Neuropsychiatric disorders [38],
- Ecology [39],
- Economics [40],
- Urbanism [41],
- Laws [42],

among others. During the latest decades, the use of Mandelbrot’s studies [43] allows scientists to propose that these systems are governed by fractal laws such as the “Constructal law” [44] or principle of least action [45,46]. Nevertheless, fractals are geometrically well described but, a general description of thermodynamics fluxes, such as Onsager’s coefficients [47,48], which generate fractality is still missing. In order to obtain a clearer physical meaning of one specific fractal system, Section 2.1 demonstrates the extent to which the entropy of one single crack can lead to fractally distributed cracks. Section 2.2 describes the relationship between entropy and seismic moment. Section 2.3 generalizes the entropy change for any system characterized by fractal geometry by using the linear nonequilibrium thermodynamics framework as the Onsager’s coefficients. Then, Section 2.4 discusses the extent of the results while the conclusion is in Section 3.

## 2. Results and Discussion 

### 2.1. Entropy of Fractals Cracks Distribution

The change of entropy $ds_{0}/dt$ required for the generation of a single crack is given by [23]:

(1) $$\frac{ds_{0}}{dt}=\frac{{\left(\mu Nv\right)}^{2}}{T^{2}}+\frac{X_{e}J_{e}}{T}$$

where μ is the coefficient of friction generated by the inner relative displacement of the crack boundaries, N the normal force, v the relative velocity, T the temperature, and $X_{e}$ and $J_{e}$ are the voltage and electrical current, respectively. Here, the first term (right side) corresponds to the frictional heating while the second one corresponds to the electrical production due the electrical imbalance in the semi-brittle plastic regime [10,11,49,50]. The generation of electromagnetic signals prior to main failure has been widely reported in laboratory experiments [3,51,52,53,54,55] and on a geodynamic scale [16,56,57,58]. By contrast, temperature changes prior to earthquakes has been poorly supported in [59]. Then, it is expected that the entropy changes are manly driven by electrical charge generation within microcracks. This implies that:

(2) $$\frac{ds_{0}}{dt}\approx \frac{X_{e}J_{e}}{T_{0}}$$

Here and after, the temperature is considered as constant. In addition, it has been shown that the electrical current generated by external uniaxial stress change is given by [60]:

(3) $$J_{e}=\frac{1}{Y_{eff}}\left(\frac{d\sigma_{u}}{dt}\right)$$

where $Y_{eff}$ is the effective Young’s modulus and $\sigma_{u}$ the applied uniaxial stress. The voltage can be obtained by using the Ohm’s law for continuum medium. That is $\vec{J}=\sigma_{e}\vec{E}$, where $\sigma_{e}$ is the electrical conductivity and $\vec{E}$ the electric field [61]. The voltage definition $X_{e}=-\int \vec{E}\cdot d\vec{a}$ [62] allows us to write the voltage as:

(4) $$X_{e}=\rho_{e}\;{\vec{J}}_{e}\cdot \Delta \vec{a}=\rho_{e}J_{e}a\cos\theta$$

where $\rho_{e}$ is the electrical resistivity and a is the distance where the electrical current flows within the crack. Using Equations (2)–(4), the entropy change for a single crack is:

(5) $$\frac{ds_{0}}{dt}\approx C_{0}J_{e}^{2}a$$

where $C_{0}=\rho_{e}\cos\theta /T_{0}$. Let us consider that a and $J_{e}$ are parallel, then $C_{0}$ is maximum. The parameter a relates the length where the electrical current flows within a single crack, it can also be considered as proportional to the volume occupied by the cracks. That is, $a=V/A_{0}$, where $A_{0}$ corresponds to an area of reference. This implies that Equation (5) is $ds_{0}/dt∼V$. This equation stands for the volume of a single crack. However, there are several cracks within a macroscopic material under uniaxial stress prior to the failure [7,8]. Then, each contribution should be considered for macroscopic material. On the other hand, it has been shown that the total entropy change of dS corresponds to the sum of all entropy change at different length scale [63]. For example, $dS=d_{nano}S+d_{meso}S+d_{macro}S$ means the nano, meso, and macro scale contribute to the total entropy change [63]. Here, cracks are also observed at different length scale. Specifically, it has been observed that the microcracks are fractally distributed [64,65]. This implies that entropy is a sum of all the volume contributions with different length scale: $dS=\sum d_{i}s\propto \sum d_{i}V$, where in a continuum fractal distribution, the entropy change turns into: dS∝∫dV=V. Then, the total change of entropy dS/dt depends on a fractal volume:

(6) $$\frac{dS}{dt}=\frac{1}{A_{0}}\underset{i}{\sum }\frac{d_{i}s}{dt}\approx \frac{C_{0}J_{e}^{2}}{A_{0}}\int_{V}dV=C_{1}{\left(\frac{d\sigma }{dt}\right)}^{2}V_{fr}$$

where $C_{1}=C_{0}/A_{0}Y_{eff}^{2}$, σ is the macroscopic uniaxial stress and $V_{fr}$ is the fractal volume defined as [66]:

(7) $$V_{fr}=\frac{4\pi^{2}}{3}\frac{D-2}{\left(3-D\right)}{\left(l_{max}\right)}^{5-D}{\left(l_{min}\right)}^{D-2}$$

where D is the fractal dimension of rocks which mainly lies between 2 and 3, $l_{min}$ is the smallest rupture radius considered and $l_{max}$ is the largest crack within the fractal distribution. For simplicity, the largest crack is considered as circular crack [17].

Experimentally, it has been shown in X-ray tomography studies on rock samples that the generation of cracks is dominated by the growth of preexisting fractures before the main failure [7]. This implies that the volume is increasing with time. In other words, $l_{max}=l_{max}\left(t\right)$. Then, Equation (6) in (7) implies that the entropy change is proportional to the growth of the largest crack $l_{max}\left(t\right)$ within the fractal volume, growth that is generated by the external stress change dσ/dt:

(8) $$dS=\Lambda \left(D\right){\left(\frac{d\sigma }{dt}\right)}^{2}l_{max}^{\left(5-D\right)}\left(t\right)dt\;\;\;\;\;$$

where $\Lambda =C_{1}\frac{4\pi^{2}}{3}\frac{D-2}{\left(3-D\right)}{\left(l_{min}\right)}^{D-2}$. Here, dS≥0 because $l_{max}$ is a distance which is positive by definition. The only manner in which dS=0 is when dσ/dt≈0 or when $l_{min}\approx 0$. The entropy change imposes that dσ/dt≠0 and $l_{min}\neq 0$. The former means that there is an input or external force required in order to generate the cracks. In other words, no stress changes, no cracks growth, and no entropy changes in the system. The latter implies that there is a lower boundary for fractality in nature. The relationship between the external forces applied by F that generates the fractal volume $V_{fr}$ and the entropy change dS can be summarized as:

(9) $$dS\propto {\left(\frac{dF}{dt}\right)}^{2}V_{fr}$$

Thus, Equations (8) and (9) indicate that the increase of entropy is obtained by the generation of tridimensional fractals in a certain domain due an increasing external force. 

#### Entropy Change in Terms of Spatial Parameters

**Figure 1:**
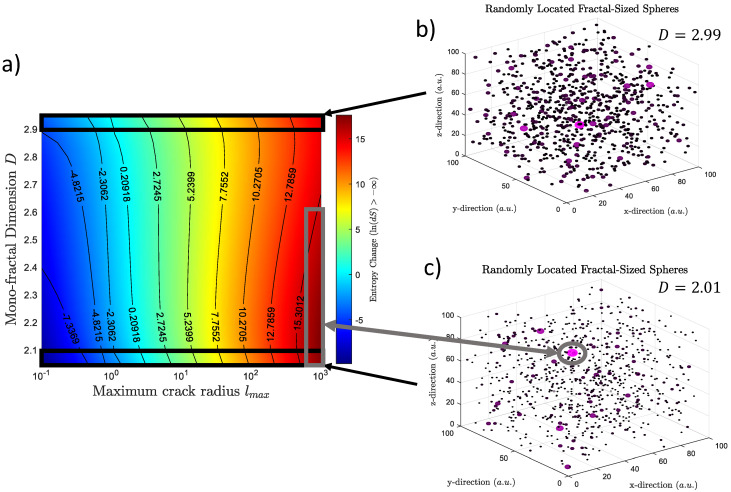
(**a**) Different values of entropy change in terms of spatial parameters. Specifically, in terms of maximum fractal length l_max and fractal dimension *D*. Note that the entropy is larger when *l_max_* is larger and *D* is smaller. (**b**) Representation of randomly located fractal volumes which are characterized by a large *D* value. (**c**) The same volume distribution but considering lower values of *D*. It is possible to observe that the volume size distribution is different when a large of smaller values of *D* are considered. When *D* is small, the domain is filled by a large number of small volume and few large volumes. The opposite is found for large *D* values.

Let us consider now a brief description of Section 2.1. Equation (8) shows the relationship between entropy and spatial properties as fractal geometry. Let us consider now how the entropy is changing in terms of specific spatial parameters as the maximum length $l_{max}$ and the fractal dimension D while dσ/dt is constant. This can be seen in Figure 1a. The entropy changes dS increases when $l_{max}$ increases regardless of the value of D. This can be seen as the change from blue to red colors in Figure 1a. Nevertheless, the dependency of dS on D is different and also dependent on $l_{max}$. For example, dS increases when D increases for small values of $l_{max}$ ($l_{max}<1$). That can be seen as the increase dS from ∼−7.3 a.u. to −4.8 a.u. in log scale. Note that a.u. means arbitrary units and the colormap of Figure 1a shows ln(dS) which allows negative values. Contrary, dS increases when D decreases for large values of $l_{max}$. The latter can be seen in the marked grey rectangle in Figure 1a. That area is characterized by a dark red color and represents large values of dS at values of $l_{max}$ close to $10^{3}$ a.u and a fractal dimension lower than 2.5. The dS values decrease for the same $l_{max}$ and large D values (D>2.5). 

Figure 1b,c shows spheres that represent the randomly distributed fractal cracks for two values of fractal dimension 2.99 and 2.01 respectively when $l_{max}$ is large. Small D shows that the domain is mainly governed by small cracks while large cracks are rare (Figure 1c). The opposite is found in (Figure 1b). 

### 2.2. Entropy Change in Terms of External Stress Change

**Figure 2:**
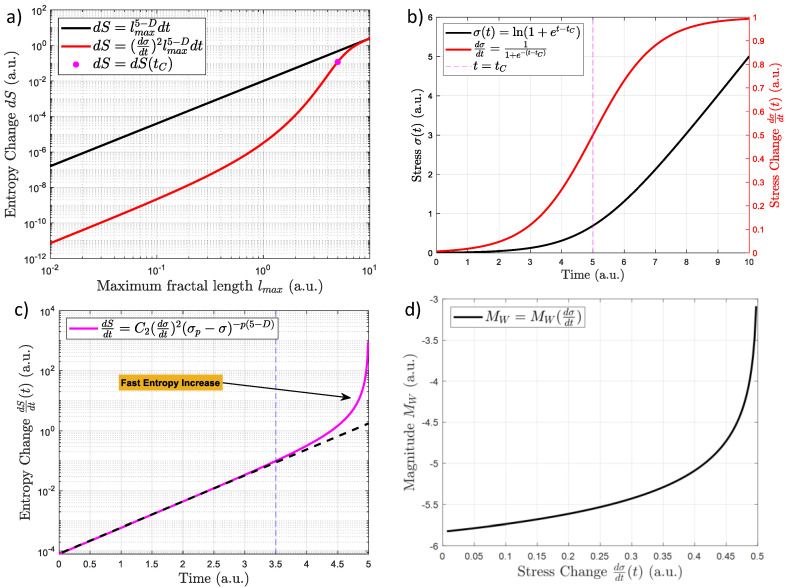
(**a**) Relation between entropy change dS and the maximum fractal crack of length $l_{max}$. The black curve shows how the entropy increases in terms of the volume growth and dσ/dt is constant. The red curve shows the incorporation of the sigmoid function in dσ/dt. The purple point shows the dS and $l_{max}$ where the earthquake occurs for the sigmoid function. (**b**) Stress evolution σ (black curve) and shear stress change dσ/dt (red curve) of the lithosphere prior to and after the main failure. Here, the earthquake time is $t=t_{C}=5$ a. u. Note that a. u. means arbitrary units. (**c**) Entropy increases by using Equation (10). It is shown that there are two main behaviors: the initial slow increase that lasts up to t∼3.5 a. u. (this trend is represented as a black dotted line) and the fast increases between 3.5 a. u. and $t_{C}=5$ a.u. (**d**) Magnitude expected in terms of the stress change.

Now it is relevant to consider if all the fractally distributed cracks increase the entropy. Let us consider two cases, one where the stress increases linearly and sigmoid-shaped in time. The first case is when dσ/dt and Λ are constant (equal to 1 arbitrary unit or a.u.). This case can be seen in the black curve in Figure 2a. Here, the entropy changes dS and $l_{max}$ increases linearly (in a log-log plot) when the fractal dimension for granite (D=2.6 ) is considered [66]. The second case is when dσ/dt could be considered as proportional to the sigmoidal shape which has been found for real earthquakes [14,15,16]. That is, $\sigma \left(t\right)∼\ln\left(1+e^{a_{s}\left(t-t_{C}\right)}\right)$ and $d\sigma /dt∼{\left(1+e^{-a_{s}\left(t-t_{C}\right)}\right)}^{-1}$ [17], where $a_{s}$ is constant (here equal to 1) and $t_{C}$ corresponds to the time where the macroscopic failure occurs. Figure 2b shows the evolution of σ (black curve) and dσ/dt (red curve) for the sigmoidal case. This input generates the entropy changes shown in Figure 2a (red curve). It is clear that the incorporation of dσ/dt reduce the entropy change for small to medium $l_{max}$ values. That is, the red curve is lower than the black one in Figure 2a. Nevertheless, in both cases, the black and red curves reach the same dS values for large $l_{max}$. Note that the vertical purple segmented line in Figure 2b shows when $t=t_{C}$. This can be seen in Figure 1a as a purple dot in red curve (Figure 2a). This shows that the incorporation of an increasing stress (dσ/dt>0) increases the entropy change dS. In other words, the external forces increase the entropy from one state to another.

**Figure 3:**
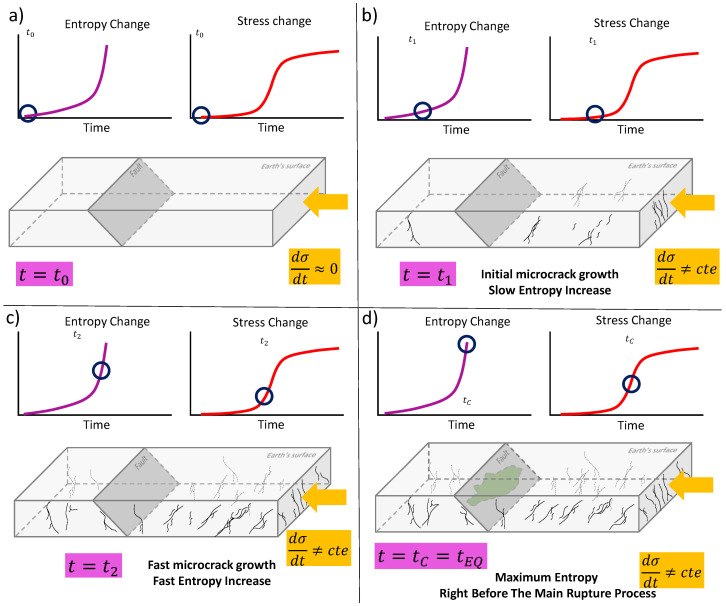
Schematic representation of the of shear stress change dσ/dt, entropy change dS/dt given by Equation (10) and the growth of microcracks prior the main failure. Initially (**a**) the almost zero dσ/dt generates no considerable stress change nor microcrack growth (blue circles). (**b**) The dσ/dt increase (red curve) is determined by a small linear increase of dS/dt (blue circles). (**c**) The fast entropy increases y related to the fast increase of the uniaxial stress. Finally, (**d**) shows that the maximum entropy change is found right before the impending main failure (marked as green area).

On the other hand, the length of correlation ξ describes the length at which the stress perturbation in cracks affect the surrounding volume [67]. It has been considered similar to the length of the largest crack during the load cycle: $\xi \propto l_{max}\;$[68,69]. This ξ is related to a second order power law for heterogeneous materials given by [69]:

(10) $$\xi =k{\left(\sigma_{P}-\sigma \right)}^{-p}$$

where $\sigma_{P}$ is the stress required for the macroscopic failure, and k and p are parameters that describe the stress evolution. Note that Equation (10) corresponds to the best fit from experiments on rock samples under compression stress. By replacing Equations (7) and (10) into Equation (6), the entropy changes of the whole system prior to the macroscopic failure is:

(11) $$\frac{dS}{dt}\approx C_{2}{\left(\frac{d\sigma }{dt}\right)}^{2}{\left(\sigma_{P}-\sigma \right)}^{-p\left(5-D\right)}$$

where $C_{2}=\Lambda k^{5-D}$. Figure 2c shows how the entropy increases by using Equation (11) and p=0.64 [69], $\sigma_{P}=5$ (a.u.) and $C_{2}=1$ a.u. Overall entropy increases prior to the earthquake. Nevertheless, it increases particularly fast from t=3.5 a.u. up to the main failure at t=5 a.u. (purple curve in Figure 2c). This means that the generation of cracks prior the macroscopic failure is part of an irreversible process which maximizes the entropy in time (see reference [70] for the relationship between maximum entropy principle and irreversibility). Figure 3 shows the schematic representation of stress evolution, crack generation, entropy increases, and final rupture. For example, Figure 3a shows the onset of the system when no stress changes are applied. That is, no cracks are generated, and entropy is at lowest value (blue circles). An increase in the stress will create a small number of cracks while the entropy slowly increases (Figure 3b). Before the main earthquake, the stress increases even more while the entropy rises faster (blue circles in Figure 3b). This state is characterized by a large number of cracks within the lithosphere. Right before the earthquake, the entropy, and crack numbers are at maximum values while the stress change is at its maximum acceleration (Figure 3d). 

#### Seismic Moment and Entropy

As the entropy is rapidly increasing before the main earthquake, let us consider the effect of Equations (8) and (11) into other seismic parameters as the earthquake’s magnitude. The ruptured area A for earthquakes can be approximate by $A=\pi l_{max}^{2}\;$[17]. Then the area in terms of the entropy from Equation (8) is:

(12) $$A=\Gamma_{0}\left(D,\frac{d\sigma }{dt}\right){\left(\frac{dS}{dt}\;\right)}^{\frac{2}{5-D}}$$

where $\Gamma_{0}\left(D,\frac{d\sigma }{dt}\right)=\pi {\left(\Lambda \left(D\right){\left(\frac{d\sigma }{dt}\right)}^{2}\right)}^{-\frac{2}{5-D}}$. On the other hand, the experimental relation between seismic moment $M_{0}$ and the rupture area is given by $A=a_{A}M_{0}^{2/3}$, where $a_{A}=1.34\times 10^{-10}\;{\left(\frac{m^{5}s}{kg^{2}}\right)}^{1/3}$ [71]. By introducing Equation (12), the seismic moment is:

(13) $$M_{0}=\Gamma \left(D,\frac{d\sigma }{dt}\right){\left(\frac{dS}{dt}\right)}^{\frac{3}{5-D}}$$

where $\Gamma =a_{A}^{-3/2}\Gamma_{0}^{3/2}$. Note that Equation (8) states that dS≥0. Here, the seismic moment is only possible if dS≠0 and dσ/dt≠0. That is: dS>0. In other words, earthquake’s occurrence requires a change of stress which generates an irreversible process that increases entropy. The Equation (13) into the seismic magnitude equation [1] gives:

(14) $$M_{W}=\frac{2}{3}\log_{10}\left(\Gamma \times {\left(\frac{dS}{dt}\right)}^{\frac{3}{5-D}}\times 10^{7}\right)-10.7$$

Equation (14) corresponds to the most probable expected magnitude of impending earthquake by regarding the entropy of the lithosphere. That is, if entropy change or stress change are known, an estimation of the expected magnitude at that moment could be obtained by using Equation (14). This can be seen in Figure 2d for the sigmoidal stress change. Note that if no earthquake occurs, it is implied that entropy or stress will keep increasing. This means that the expected magnitude will also increase. 

### 2.3. Entropy and Fractal Geometry Generalization for Linear Nonequilibrium Thermodynamics 

Let us consider now the generalization for Equations (8) and (9). The first step is to consider the entropy change in time which is defined as [72]:

(15) $$\frac{dS}{dt}=\overset{N}{\underset{k=1}{\sum }}X_{k}J_{k}$$

where $X_{k}$ and $J_{k}$ are the N thermodynamics forces and flows in the system. These forces and flows are related by the phenomenological equations which are defined as:

(16) $$X_{k}=\overset{n}{\underset{l=1}{\sum }}K_{kl}J_{l}\;J_{k}=\overset{n}{\underset{l=1}{\sum }}L_{kl}X_{l}$$

where $K_{kl}$ and $L_{kl}$ are the phenomenological coefficient. Specifically, the resistance and conductance coefficients respectively. The phenomenological coefficient obeys the Onsager’s relations when no external magnetic field or Coriolis force are present [47,48]:

(17) $$L_{kk}>0\;\;\;\;\left(k=1,2,\ldots ,n\right)$$

The condition from Equation (17) is satisfied because the magnetic anomalies are the results of the crack generation. Magnetism is not generating or affecting the lithospheric stress states as shown in Equation (3). Then, Equation (17) for different indices becomes:

(18) $$L_{ll}L_{kk}>\frac{1}{4}{\left(L_{il}+L_{li}\right)}^{2}\;\;\;\left(l\neq k;k,l=1,2,\ldots ,n\right)$$

Additionally, the matrix $L_{kl}$ and $K_{kl}$ are related by:

(19) $$K=L^{-1}$$

Let us consider the entropy change $dS^{\mu }/dt$ for different subdomain  μ (μ=1,2,…,m). Then, Equation (15) becomes:

(20) $$\frac{dS^{\mu }}{dt}=\overset{N}{\underset{k=1}{\sum }}X_{k}^{\mu }J_{k}^{\mu }$$

and the thermodynamic forces and flows are now expressed as:

(21) $$X_{k}^{\mu }=\overset{n}{\underset{l=1}{\sum }}K_{kl}^{\mu }J_{l}^{\mu }\;J_{k}^{\mu }=\overset{n}{\underset{l=1}{\sum }}L_{kl}^{\mu }X_{l}^{\mu }$$

Replacing the forces from Equation (21) into (20) gives:

(22) $$\frac{dS^{\mu }}{dt}=\overset{N}{\underset{k=1}{\sum }}\overset{n}{\underset{l=1}{\sum }}K_{kl}^{\mu }J_{l}^{\mu }J_{k}^{\mu }$$

Here, the phenomenological coefficient cannot be negative while flows (or forces) are quadratic in forms which implies that $dS^{\mu }/dt\geq 0$ [73]. Equations (16) and (21) show that all the forces $X_{i}$ can be generated by all the flows $J_{i}$ (and vice versa) by a linear combination of the resistance (or conductance) coefficients. For example, Equation (4) shows that the proportionality between electrical currents and volts holds when the distance at which the volt is considered is not zero. In other words, the phenomenological coefficients are related to the domain’s length for this case. Similarly, thermal conductivity depends on the Knudsen number which depends on the characteristic length [74,75,76] while the hydraulic conductivity is proportional to the hydraulic permeability which is a measure of the pore geometry of the pore structure [77]. In addition, the resistivity K can be related to the local metric tensor which represents the geometric measurement of the distance traveled [78]. Other examples of the relation among thermodynamics forces, flows, and phenomenological coefficients that require geometrical properties as volume or characteristic length can be seen in Table 3.1 in reference [79]. The above-mentioned examples suggest that phenomenological coefficients $K_{kl}^{\mu }$ describe the geometrical properties as the characteristic length $r_{kl}^{\mu }$ which defines those domains where $X_{k}^{\mu }$ and $J_{k}^{\mu }$ are valid. This means that the phenomenological coefficients can be written as: $K_{kl}^{\mu }=\eta_{0}K^{\;}\prime_{kl}^{\mu }{\left(r_{kl}^{\mu }\right)}^{\alpha }r_{0}^{-\alpha }$, where $\eta_{0}$ is dimensionless constants, $r_{0}$ a constant of units of length, $K^{\;}\prime_{kl}^{\mu }$ correspond to a constant with the phenomenological coefficient units, and α is a constant that determine the dimension of the characteristic length r. That is, α represents Euclidian dimensions or fractal dimensions. Then, the total entropy change is:

(23) $$\frac{dS}{dt}=\overset{m}{\underset{\mu =1}{\sum }}\frac{dS^{\mu }}{dt}=\eta_{0}\overset{m}{\underset{\mu =1}{\sum }}\left\{\overset{N}{\underset{k=1}{\sum }}\overset{n}{\underset{l=1}{\sum }}{\left(r_{kl}^{\mu }\right)}^{\alpha }\frac{K\prime_{kl}^{\mu }J_{l}^{\mu }J_{k}^{\mu }}{r_{0}^{\alpha }}\;\right\}$$

In other words, the total entropy depends on the entropy of each subdomain ($dS/dt=dS/dt\left(dS^{\mu }/dt\right)$, (μ=1,2,…,m)). On the first hand, a general self-affine transformation between a pair of nonoverlapped subdomains $M^{\mu }$ and $M^{\prime \mu }$ (μ=1,2,…,m) is governed by the relation $M^{\prime \mu }=\xi M^{\mu }$, where

(24) $$\xi ={\left[\begin{array}{cccc} \xi_{11} & 0 & \cdots & 0\\ 0 & \xi_{22} & \ldots & 0\\ \vdots & \vdots & \ddots & \vdots \\ 0 & 0 & \cdots & \xi_{mm} \end{array}\right]}_{m\times m}\;$$

And $\xi_{11},\xi_{22},\ldots ,\xi_{\mu \mu }$ are constants that represent dilatancy ($\xi_{\mu \mu }>1$) or contractions ($\xi_{\mu \mu }<1$) among different subdomains $M^{\mu }$ [80]. Here, Equation (24) represents the diagonal self-affinity when all the terms of the diagonals are equals. That is, when $\xi_{11}=\xi_{22}=\ldots =\xi_{\mu \mu }=\ldots =\xi_{mm}=\xi_{0}$. Then, the self-affine transformation is given by: $(M^{\prime 1},M^{\prime 2},\ldots ,M^{\prime \mu },\ldots ,M^{\prime m})=\left(\xi_{11}M^{1},\xi_{22}M^{2},\ldots ,\xi_{\mu \mu }M^{\mu },\ldots ,\xi_{mm}M^{m}\right)=\xi_{0}\left(M^{1},M^{2},\ldots ,M^{\mu },\ldots ,M^{m}\right)$. In other words, each subdomain $M^{\prime \mu }$ corresponds to a larger or smaller version of other subdomain $M^{\mu }.$ Mathematically, this scale invariance can be written as [80]:

(25) $$g\left(\lambda ,x\right)=\lambda^{\alpha_{0}}g\left(x\right)$$

where λ is a factor and $\alpha_{0}$ is a constant. To consider our system as self-affine, each length $\xi_{\mu \mu }$ must be constant and characterize the length of each subdomain μ. This means that if the self-affine property is applied to Equation (23), it implies that $r_{kl}^{\mu }$ does not depend on indices k and l. In other words, the N net forces and flows of each subdomain μ are restricted within a specific length scale ${\left(r^{\mu }\right)}^{\alpha }$. Thus, the latter turn Equation (23) into:

(26) $$\frac{dS}{dt}=\overset{m}{\underset{\mu =1}{\sum }}\frac{dS^{\mu }}{dt}=\eta_{0}\overset{m}{\underset{\mu =1}{\sum }}{\left(r^{\mu }\right)}^{\alpha }\left\{\overset{N}{\underset{k=1}{\sum }}\overset{n}{\underset{l=1}{\sum }}\frac{K\prime_{kl}^{\mu }J_{l}^{\mu }J_{k}^{\mu }}{r_{0}^{\alpha }}\;\right\}=\eta_{0}\overset{m}{\underset{\mu =1}{\sum }}{\left(r^{\mu }\right)}^{\alpha }\overset{N}{\underset{k=1}{\sum }}{\hat{X}}_{k}{\hat{J}}_{k}=\eta_{0}\overset{m}{\underset{\mu =1}{\sum }}{\left(r^{\mu }\right)}^{\alpha }\frac{d{\hat{S}}^{\mu }}{dt}$$

where ${\hat{X}}_{k},\;{\hat{J}}_{k}$, and ${\hat{S}}^{\mu }$ are defined as the thermodynamics forces density, thermodynamics flows density, and entropy change density and include the quadratic forces. Then, it is possible to observe from 26 that $f\left(\lambda ,r\right)=\frac{dS}{dt}\left(\lambda ,r\right)=\lambda^{\alpha }\frac{dS}{dt}\left(r\right)=\lambda^{\alpha }f\left(r\right)$ which satisfy the scale invariance definition from Equation (25) and where $\alpha_{0}=\alpha$. In the continuum self-affine limit, the total entropy is:

(27) $$\frac{dS}{dt}=\eta_{0}\int_{r_{min}}^{r_{max}\;}\frac{d{\hat{S}}^{\;}}{dt}r^{\alpha }dr$$

On the second hand, a general fractal geometrical volume $V_{E}$ is obtained when $V_{E}∼\int r^{D_{E}}dN$, where $N=k_{1}r^{-D}$, $k_{1}$ is a constant $D_{E}$ is the Euclidian dimension, and D is the fractal dimension [66]. This gives the relation: $V_{E}∼-Dk_{1}\int r^{D_{E}-D-1}dr$. Then, the entropy is fractally distributed when $\alpha =D_{E}-D-1$. In other words, Equation (27) is a generalization of the relation $dS/dt=\int_{V}dS_{v}/dtdV$ used in reference [73], where $S_{v}$ is the entropy density (chapter 3.9.1). If the entropy change density is fractally distributed, the contributions are only a dilated or contracted version of a constant $d{\hat{S}}_{0}/dt\;$(Equation (26). Thus, the contribution of entropy change of Equation (27) is independent of r. Thus, the total entropy change is:

(28) $$\frac{dS}{dt}=-\eta_{0}Dk_{1}\frac{d{\hat{S}}_{0}{}^{\;}}{dt}\int_{r_{min}}^{r_{max}\;}r^{D_{E}-D-1}dr=-\frac{\eta_{0}Dk_{1}}{D_{E}-D}\frac{d{\hat{S}}_{0}{}^{\;}}{dt}\left[r_{max}^{D_{E}-D}-r_{min}^{D_{E}-D}\right]$$

The constant $k_{1}$ can be found by using the topological dimension $D_{T}$ that obeys $A_{T}=-Dk_{1}\int r^{D_{T}-D-1}dr$, where $A_{T}$ is a known positive constant. Then, the total entropy change is:

(29) $$\frac{dS}{dt}=\frac{d{\hat{S}}_{0}{}^{\;}}{dt}\frac{\eta_{0}A_{T}\left(D_{T}-D\right)}{\left(D_{E}-D\right)}\frac{\left[r_{max}^{D_{E}-D}-r_{min}^{D_{E}-D}\right]}{\left[r_{max}^{D_{T}-D}-r_{min}^{D_{T}-D}\right]}$$

Fractal dimension values range from $D_{T}<D<D_{E}$ [43] which implies that $D_{E}-D>0$ and $D_{T}-D<0$. This also implies that $\left(D_{T}-D\right)/\left(D_{E}-D\right)<0$. Note that $\frac{dS}{dt}=0$ if $D=D_{T}$. For fractal, it is also valid that: $r_{max}^{D_{E}-D}\gg r_{min}^{D_{E}-D}$ and $r_{max}^{D_{T}-D}\ll r_{min}^{D_{T}-D}$. These results applied to Equation (29) show that the geometrical part of Equation (29) is always positive. Then, Equation (29) becomes:

(30) $$\frac{dS}{dt}\approx \frac{d{\hat{S}}_{0}{}^{\;}}{dt}\frac{\eta_{0}A_{T}\left(D-D_{T}\right)}{\left(D_{E}-D\right)}\left(r_{max}^{D_{E}-D}\right)\left(r_{min}^{D-D_{T}}\right)$$

As Equation (22) is defined always as nonnegative, Equations (29) and (30) can be written in a more general manner as:

(31) $$\frac{dS}{dt}\approx \frac{d{\hat{S}}_{0}}{dt}\Phi_{V}\left(r_{max},D_{T},D,D_{E}\right)\geq 0$$

where $\Phi_{V}=\frac{\eta_{0}A_{T}\left(D-D_{T}\right)}{\left(D_{E}-D\right)}\left(r_{max}^{D_{E}-D}\right)\left(r_{min}^{D-D_{T}}\right)$. Here a fixed $r_{min}$ is used. Note that Equation (6) is recovered if we consider the volumetric contribution V by using the Euclidean dimension $D_{E}=3$, $D_{T}=D_{E}-1,$ $\eta_{0}=4\pi /3$ and $A_{T}=\pi r_{max}^{2}$. While the quadratic forces are represented by $d{\hat{S}}_{0}/dt$ as shown in Equation (26).

Finally, it is possible to observe that the volume part V in Equation (31) grows when the larger fractal structure length $r_{max}=r_{max}\left(t\right)$ is larger. In other words the larger the fractal volume, the larger the entropy increases. 

#### Multifractal Entropy for Linear Nonequilibrium Thermodynamics 

Equation (31) depends on the fractal dimension D. Nevertheless, D is not a fixed value because there could be infinite D values between $D_{T}$ and $D_{E}$. This raises the possibility to take into account a system composed by subdomains characterized by different fractal dimensions. If each subdomain is restricted to a specific volume, it is possible to consider the volume growth of several non-interacting fractal structures by adding k different entropy contribution. That is:

(32) $$\frac{dS}{dt}\approx \frac{d{\hat{S}}_{0}}{dt}\Phi_{V_{0}}\left(r_{max_{0}},D_{T_{0}},D_{0},D_{E_{0}}\right)+\frac{d{\hat{S}}_{1}}{dt}\Phi_{V_{1}}\left(r_{max_{1}},D_{T_{1}},D_{1},D_{E_{1}}\right)+\ldots +\frac{d{\hat{S}}_{k}}{dt}\Phi_{V_{k}}\left(r_{max_{k}},D_{T_{k}},D_{k},D_{E_{k}}\right)\geq 0$$

Equivalently:

(33) $$\frac{dS}{dt}\approx \underset{k}{\sum }\frac{d{\hat{S}}_{k}}{dt}\Phi_{V_{k}}\geq 0$$

Then, the contribution of several non-interacting fractals volumes’ also increase the total entropy of the system. The interacting systems case is different. 

### 2.4. Discussion

Cracks and fractures within the lithosphere are well described by scaling laws or fractals distributions [81]. This implies that experiments on rock samples could also give information regarding the geodynamic scale. One relevant property of rock samples is the increasing number of cracks before the main failure [7,8,54,69]. This means that the role of cracking and its electromagnetic signals correspond to a pre-failure feature that can be used as a forecast for major earthquakes [4]. On the other hand, it is known that the lithospherical surface deformation is a feature of the interseismic cycle which is observed in the middle of two large earthquakes [82]. Then, the link between interseismic and the pre-failure process could be stated as it follows: the surface deformation is the first reaction to the stress increases while the cracking generation rises when the lithosphere cannot hold more strain. That is, the cracking is generally generated when no deformation is clearly observed. Up to some point, the strain and cracking will not be sufficient to hold the still-increasing stress. It is at this moment when the main earthquake is expected to occur. 

Here, as Equation (8) shows that the crack generation rapidly increases the entropy, it could be considered that the fractally distributed cracking process corresponds to the main manifestation of lithospheric dynamics despite the not clearly observed deformation. Note that large scale strain-rate perturbation still could exist [83]. As the main earthquake is also a manifestation of entropy increases, the entropy change can be used in order to link the pre-failure dynamic to the main failure (or crack) in order to estimate an approximate magnitude of the impending earthquake. This can be done by considering the Equations (13) and (14). These Equations show that the larger the entropy (or stress) change, the larger the expected earthquake magnitude. In addition, Equations (13) and (14) also state that no earthquake can be expected if there is no entropy (stress) change. In other words, both the main rupture and crack generation are linked by the entropy increases. This means that the earthquake process must be preceded by the crack generation within the lithosphere that also could generate electromagnetic signals. This contradicts those claims that consider the space weather and solar activity as the main source of pre-failure electromagnetic signals [84]. Despite this, there is one manner in which space weather could influence the lithospherical cracking. That is, by means of external forces. Specifically, part of the stress changes might be generated by solar activity. This also implies that a reliable space weather mechanism must be presented. Otherwise, if no relation between solar activity and lithospherical stress changes is confirmed, implies that the cracking process required to increase the lithospherical entropy is driven only by tectonic forces. 

**Figure 4:**
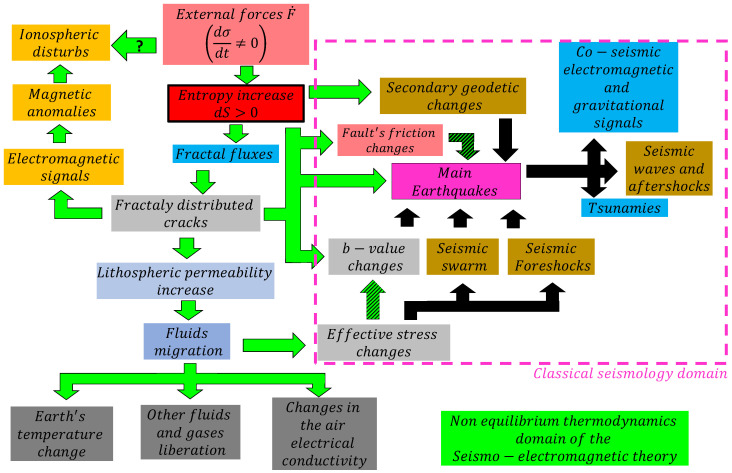
Schematic representation of the seismo-electromagnetic domain. It is possible to observe that the cascade of physical phenomena start with stress changes that increase the entropy of the lithosphere. Then, the green arrows represent the seismo-electromagnetic branch that explain the observed seismic and non-seismic pre-earthquake measurements. The black arrows represent the classical seismic domain. Note that the physics that explain earthquake occurrences come from the seismo-electromagnetic domain. That is, the change of b-value, the main earthquake, and secondary effects and fault’s frictional changes are due the entropy increases. Those green-black arrows represent relation that can be stablished by classical seismology and seismo-electromagnetic phenomena.

As those tectonic forces increase the number of cracks, it is implied that they also increase the interaction between fractures and pores which leads to the increase of the fracture permeability [85]. In addition, the Darcy’s law states that the larger the permeability, the larger the fluid flow within the media [86]. This means that the increase of fractal cracks imply the increase of the permeability, which also allows fluid migration within the lithosphere. This fluid migration disturbs the lithospheric effective stresses [87] which could destabilize stress states within faults [88]. Additionally, the fluid migration could also flow upward the Earth’s surface while carrying high temperatures, different gases, and electric charges that could ionize the lower atmosphere [89,90,91,92]. The latter physical description can be added to the cascade of physical processes that rise due the entropy increases. The whole schematic representation can be seen in Figure 4. Here, the main physical property that triggers the cascade of other physical phenomena is the stress increase and the increase of the entropy. The green arrow represents the different process that can be explained by the seismo-electromagnetic theory when non-equilibrium thermodynamics is considered. This includes the fractal cracking, electromagnetic signals, fluids migration, frictional changes, b-value changes, main earthquake generation, and other less direct processes such as gases liberation [93], electrical charge movement [94], or ionospheric anomalies (see Figure 10 in reference [89] for the cascade of physical process considered as not direct effect). The black arrows show the classical seismological relations such as tsunamis, aftershocks, and gravitational signals. Figure 4 shows that the seismo-electromagnetic theory in the context of non-equilibrium thermodynamics complements classical seismology and gives a multidisciplinary physical explanation of the earthquake’s generation. Note that this description is regardless of the geological context because all of these cascades of phenomena have been reported in different seismic events and rises because of the cracking of intact rock [95,96,97].

Regarding the geometrical distribution of cracks, this is a key feature that allows us to obtain Equations (8), (13), and (14). This is the same fractal distribution which is also observed in other fields under other names as “Constructal law” [44,98]. That is why Equations (29)–(31) were obtained in Section 2.3 and correspond to the generalization of Equation (8). Specifically, it is shown how geometrical properties of the phenomenological coefficients (Onsager’s relations), which relates thermodynamics forces and flows, can also be valid for fractal distribution (Equations (29)–(31) in Section 2.3). Note that Equations (31) and (33) also share the shape shown in Equation 9. Here, the terms $d\hat{s}/dt$ represent the quadratic time derivative force ($d\hat{s}∼{\dot{F}}^{2}$). 

Equations (29)–(31) also show the general relation between the growth of a fractal distribution and the increase of entropy in time. By considering this, it is possible to claim that the growth of fractals in nature correspond to the rise of one kind of irreversible ‘disorder’ governed by Equations (29)–(31).

It is also important to note the link between the Constructal law, the Onsager’s relations, the metric tensor, and multiscale thermodynamics. As the Constructal Law describes the energy flux of natural systems that are characterized by fractal geometry [99], and the Onsager’s coefficients describe the thermodynamic flux in non-equilibriums systems (Equation 16), it is possible to state that the Constructal law is equivalent to the phenomenological coefficients when time-dependent fractal geometry is considered. Furthermore, as the metric tensor is related to the Onsager’s resistivity K [78], and the fractal volume in Equations (31) and (33) scales K (Equation (26)), which implies that the metric tensor could have fractal or multiscale properties. This also implies that future works should relate this fractal entropy (Equation (32)) to the multiscale thermodynamics [100] or even cosmological evolution [101] and quantum gravity [102,103]. This is because these kinds of links would allow other deepest question to be asked, such as: are fractals the results of random fluxes and forces or the results of space time properties? Are those forces and fluxes being shaped by the fractal metric tensor? Are fractals the milestone required to link quantum and gravity realms? More work must be done in this direction. 

## 3. Conclusions

This work has described the thermodynamics of fractals cracks presented in the seismo-electromagnetic theory prior to main earthquakes. This example has been useful in order to generalize the thermodynamics of systems characterized by fractal geometries. Regarding the fractal cracking process, it is possible to conclude that:

- As Equation (8) is always positive, it is implied that the generation of cracks are the manifestation of irreversible process.
- The pre-failure and failure process can be linked by means of the entropy changes.
- The seismic moment and magnitude exist if external stress, that increases of the entropy of the lithosphere, and increases in the number of cracks and electromagnetic signals also exist.
- It is possible to estimate an expected seismic magnitude in terms of the entropy change/stress change.
- Entropy rapidly increases before earthquakes.
- No entropy increase, no earthquake.
- The seismo-electromagnetic theory explains the non-seismic pre-earthquakes signals and gives physical foundations to the generation of earthquakes.

Regarding the generalization of other non-equilibrium system characterized by fractal properties, it can be concluded:

- The tendency in which nature creates fractals corresponds to a geometrical manifestation of that tendency in which the universe increases the entropy.
- Fractals rising in several fields and topics reveals the increase of ‘disorder’ of those systems.
- The phenomenological coefficients can describe geometrical properties of forces and fluxes.
- The Constructal law is one geometrical application of Onsager’s relations.
- The entropy density is defined as $d\hat{s}$, which represents the quadratic time derivative of those forces (${\dot{F}}^{2}$) that generate the fractal geometry $V_{fr}$. No changing force F implies no fractality.
- More work must be done in order to link metric tensor, fractal entropy and multiscale thermodynamics.

## Data Availability

Not applicable.
